# Softened Microstructure and Properties of 12 μm Thick Rolled Copper Foil

**DOI:** 10.3390/ma15062249

**Published:** 2022-03-18

**Authors:** Rui Feng, Weichao Zhao, Yumei Sun, Xiaowen Wang, Benkui Gong, Baoping Chang, Tianjie Feng

**Affiliations:** 1 School of Materials Science and Engineering, Shandong University of Technology, Zibo 255000, China; fengrui@sdut.edu.cn (R.F.); zhaoweichao1998@163.com (W.Z.); sunyumei4243@163.com (Y.S.); lvhupo@126.com (X.W.); 2 Heze Guangyuan Copper Strip Co., Ltd., Heze 274009, China; gycf@vip.sina.com (B.C.); gycs@vip.163.com (T.F.)

**Keywords:** rolled copper foil, microstructure, texture, properties, electrical properties

## Abstract

Up to now, 12 μm thick rolled copper foil is the thinnest rolled copper foil that can be stably produced. The softened microstructure and properties of 12 μm thick rolled copper foil were systematically studied in this paper. The softened process consists of thermal treatment at 180 °C for different times. The results show that the softened annealing texture is mainly cubic texture, and the cubic texture fraction increases with the increase in annealing time. The cubic texture fraction reaches the highest (34.4%) after annealing for 60 min. After annealing for 1–5 min, the tensile strength and the bending times decrease significantly. After annealing for 10–60 min, the tensile strength tends to be stable, and the bending times increase slightly. With the increase in annealing time, the electrical conductivity increases gradually, reaching 92% International Annealed Copper Standard (IACS) after annealing for 60 min. Electrical conductivity can be used as a fast and effective method to analyze the microstructure of metals.

## 1. Introduction

Rolled copper foil is widely used in the fields of flexible printed circuit board, lithium-ion battery, artificial intelligence, aerospace, etc. [1,2,3]. With the development of being miniaturized, lightweight, high speed and multi-functional in electronic and electrical components, higher requirements are put forward for the processing quality [4]. The etching processing is the key technology used to prepare high-end circuit board, especially for the fine flexible printed circuit board with the line width and line distance of no less than 30 μm [5,6]. The etching processing is an unstressed processing method. Firstly, the photoresist is used to protect the part of the workpiece, then, a strong oxidant is used to etch the other parts, and finally, the required components are obtained [7]. The etching processing has strict requirements on the internal stress, warpage, surface quality and etching performance of rolled copper foil [8,9].

With the continuous development of electronic products and the improvement of etching performance requirement, higher requirements are put forward for the state of rolled copper foil of fine flexible printed circuit board [10]. At present, the 180 °C softened rolled copper foil is mainly used in the field of high-end flexible printed circuit board, while the rolled copper foil without softened annealing cannot be used in this field [11]. Additionally, the flexible printed circuit board requires good bending resistance performance [12]. Previous research has shown that the uniform grain orientation reduces the stress concentration at the grain boundary, which is helpful to improve the bending resistance performance of copper foil [13,14]. Obtaining uniform grain orientation by softened annealing is the basis of improving the bending resistance performance of rolled copper foil.

Therefore, the softening of copper foil has a very important influence on its etching and bending resistance performance. The deformation rate of 12 μm thick rolled copper foil is more than 95%, which leads to the existence of large internal stress. So far, 12 μm thick rolled copper foil is the thinnest rolled copper foil that can be stably produced. In order to further improve its etching performance, especially the bending resistance performance, the research on the softened microstructure and properties has gradually attracted the attention of scholars [15]. In addition, for the 12 μm thick rolled copper foil, creating a good sample for microstructure observation and properties testing is a challenging task. In this paper, the softened microstructure and properties of 12 μm thick rolled copper foil were systematically studied.

## 2. Experimental Materials and Methods

The experimental materials are samples of 12 μm thick rolled copper foil (Ag ≤ 0.01%) produced by a domestic company (Heze Guangyuan Copper Strip Co., Ltd., Heze, China). They were annealed at 180 °C for 1 min, 3 min, 5 min, 10 min, 15 min, 30 min and 60 min in 101-0BS electric blast drying oven (Shanghai Lichen Instrument Technology Co. Ltd., Shanghai, China). Before surface observation and analysis, the samples were electropolished in 60% *w*/*w* H_3_PO_4_ with *U* = 15 V for 60 s, followed by rinsing with tap water and deionized water to remove any amorphous film formed during the electropolishing process. Then, the samples were processed by the IM4000 Hybrid Ion Milling System (Hitachi, Tokyo, Japan). The values of discharge voltage, ion beam irradiation angle, specimen rotation speed, specimen rotation speed and Ar gas flow were 1.5 kV, 4°, 80 r/min, 25 r/min, 1 cm^3^/min, respectively. After the electropolishing and ion grinding, the annealed copper foil was observed by FEI-Apreo Field Emission Scanning Electron Microscope (FESEM, FEI, Waltham, MA, USA), Tecnai G2F 20 Field Emission Transmission Electron Microscope (FETEM, FEI, Waltham, MA, USA) and analyzed by electron backscatter diffraction (EBSD, FEI, Waltham, MA, USA). The HKL Channel 5 system was adopted in the EBSD data analysis, such as the grain orientation, texture type and grain boundary type. The conductivity, tensile properties and bending properties were tested by FT-330 Four Probe Resistivity Tester (Ningbo Ruike Weiye Instrument Co. Ltd., Ningbo, China), Instron 5965 universal material testing machine (Instron, Norwood, MA, USA) and ASIDA-NIR bending tester (Guangdong Zhengye Technology Co. Ltd., Dongguan, China).

## 3. Results and Discussion

### 3.1. Microstructure

**Figure 1:**
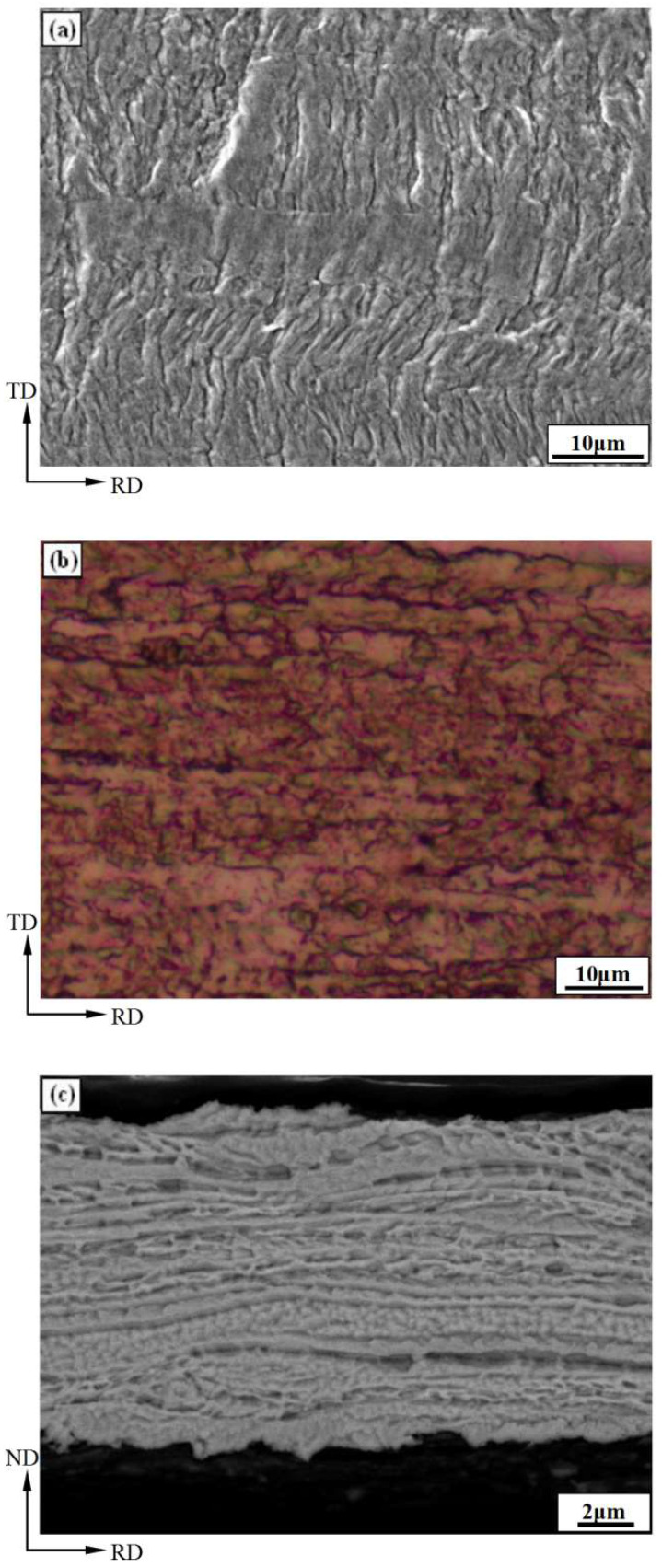
Morphology of copper foil surface and longitudinal section. (**a**) Surface morphology (SEM); (**b**) Surface morphology after corrosion (OM); (**c**) Longitudinal section morphology (SEM).

Figure 1 shows the micro-morphology of the surface and longitudinal section of the rolled copper foil. Figure 1a,b exhibit the surface morphology observed by SEM and the surface morphology after corrosion observed by optical microscope (OM), respectively. It can be seen that the surface of rolled copper foil is relatively rough, and its *R_a_* and *R_z_* values are 0.10 μm and 0.80 μm, respectively. Meanwhile, there are a lot of rolling traces on the surface, and the length direction of rolling traces is perpendicular to the rolling direction (RD). The surface grains are elongated along the RD orientation, as shown in Figure 1b,c, which exhibit the SEM morphology of the longitudinal section. It can be seen that the grains are elongated along the RD orientation to form fibrous grains through the whole thickness direction, and its length direction of grains is perpendicular to the transverse direction (TD), which indicates that the copper foil has undergone large plastic deformation.

**Figure 2:**
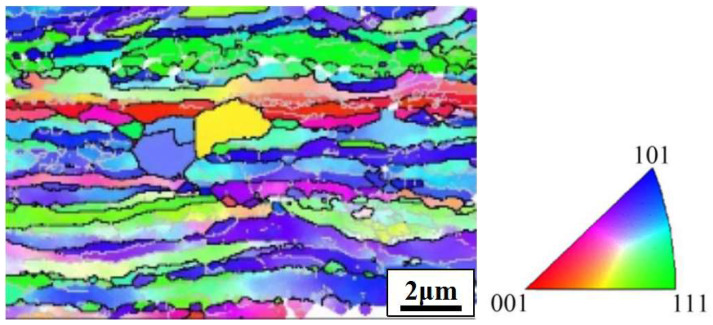
IPF diagram of EBSD orientation image of the longitudinal section of copper foil.

Figure 2 shows the inverse pole figure (IPF) diagram of EBSD orientation image of the longitudinal section of rolled copper foil. It can be seen that the grains keep the deformation grain characteristics along the rolling direction, which are mainly <111> and <101> orientations. This is because the copper is a face-centered cubic (fcc) metal, and the deformation results from the dislocation slip. The <111> and <101> orientations are stable orientations, and the grain orientation will continuously transfer to these two orientations during rolling.

**Figure 3:**
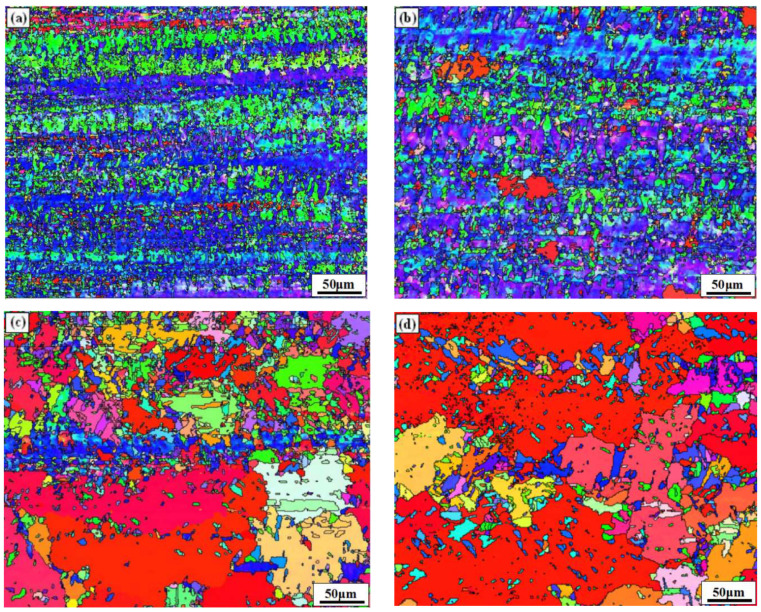

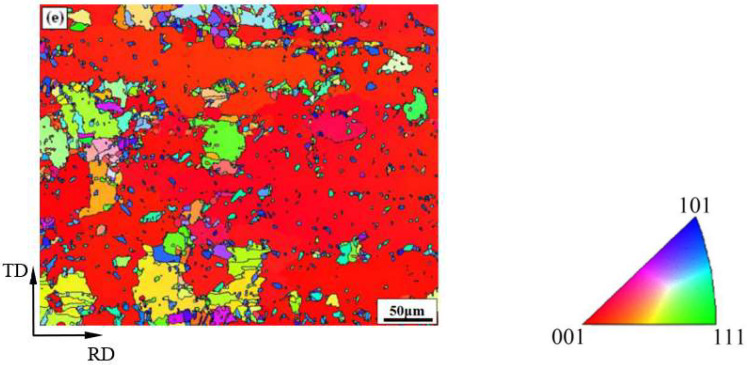
IPF diagram of the rolled and annealed copper foil. (**a**) Rolled sample; (**b**) 1 min; (**c**) 5 min; (**d**) 10 min; (**e**) 60 min.

Figure 3 exhibits the IPF diagram of the surface of the rolled and annealed copper foil. It can be seen from Figure 3a that the surface grains of the rolled copper foil are crushed and refined by large deformation rate rolling, and the grain orientation is banded along the RD orientation. After annealing for 1 min, a small amount of fine equiaxed grains (red grains in Figure 3b) appears on the surface, indicating that the local region of the copper foil begins to recrystallize. After annealing for 5 min, a small amount of deformed grains (blue grains in Figure 3c) remains on the surface. After annealing for 10 min (Figure 3d), the deformed fibrous grains disappear, and complete recrystallization occurs. After annealing for 60 min (Figure 3e), the grains grow into equiaxed grains.

**Figure 4:**
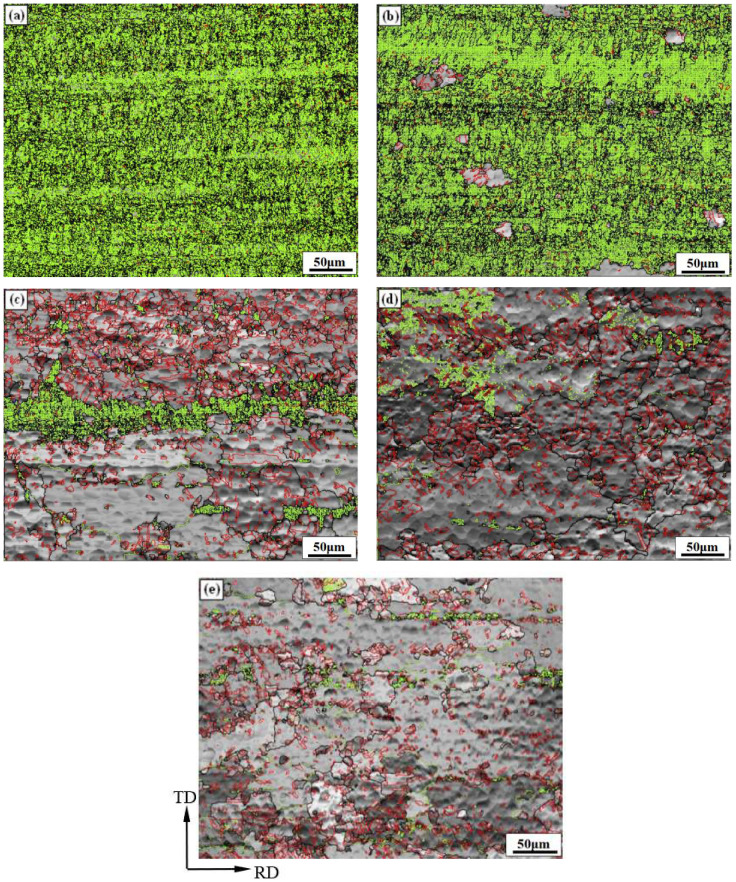
Grain boundary distribution of rolled and annealed copper foil. (**a**) Rolled sample; (**b**) 1 min; (**c**) 5 min; (**d**) 10 min; (**e**) 60 min.

**Figure 5:**
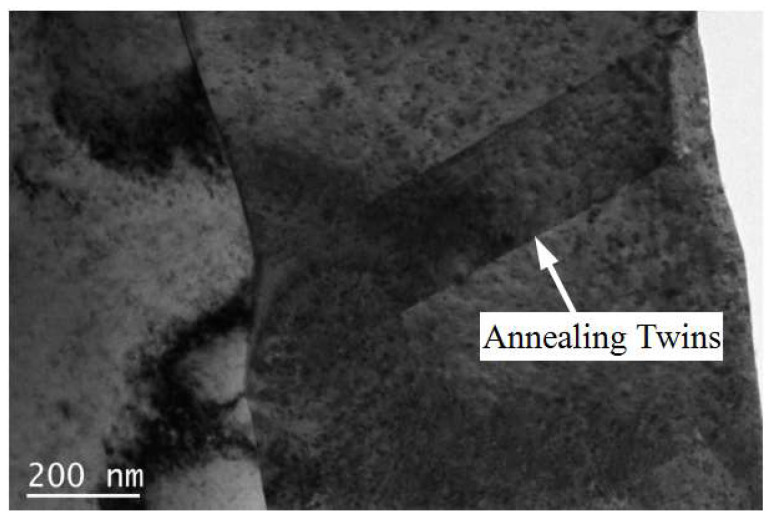
Annealing twins formed during annealing process observed by TEM.

The grain boundaries, especially the grain boundaries’ angle of deformed microstructure, are the basis of analyzing the recovery and recrystallization during the subsequent annealing process [16]. Because of the symbiotic relationship between annealing twins and recrystallized structure [17], Σ3 grain boundaries are annealing twin boundaries, which can be used as the sign to reflect the recrystallization of rolled copper foil during the annealing process. Figure 4 shows the grain boundary distribution of rolled and annealed copper foil, in which the green line represents low angle grain boundaries (LAGBs, 2 ≤ *θ* ≤ 15°), the black line represents high angle grain boundaries (HAGBs, *θ* > 15°), and the red line represents Σ3 grain boundaries. Table 1 shows the fraction of different grain boundary types. It can be seen that the fraction of LAGBs of the rolled copper foil is 68.5%. After annealing for 1 min, the fraction of LAGBs and HAGBs changes little compared with that of rolled copper foil, and the fraction of Σ3 grain boundaries increases slightly. After annealing for 5 min, the fraction of LAGBs decreases sharply, while the fraction of HAGBs increases significantly. Meanwhile, the fraction of Σ3 grain boundaries reaches 47.5%, which indicates that a large number of annealing twins have formed during the annealing process, as shown in Figure 5. After annealing for 10 min, the fraction of Σ3 grain boundaries increases to 63.3%. After annealing for 60 min, the fraction of Σ3 grain boundaries decreases slightly. With the increase in annealing time, the fraction of HAGBs increases. The fraction of Σ3 grain boundaries increases at the recrystallization stage, while the fraction decreases at the grain growth stage. When annealed for 10 min, the fraction of Σ3 grain boundaries reaches the highest.

**Figure 6:**
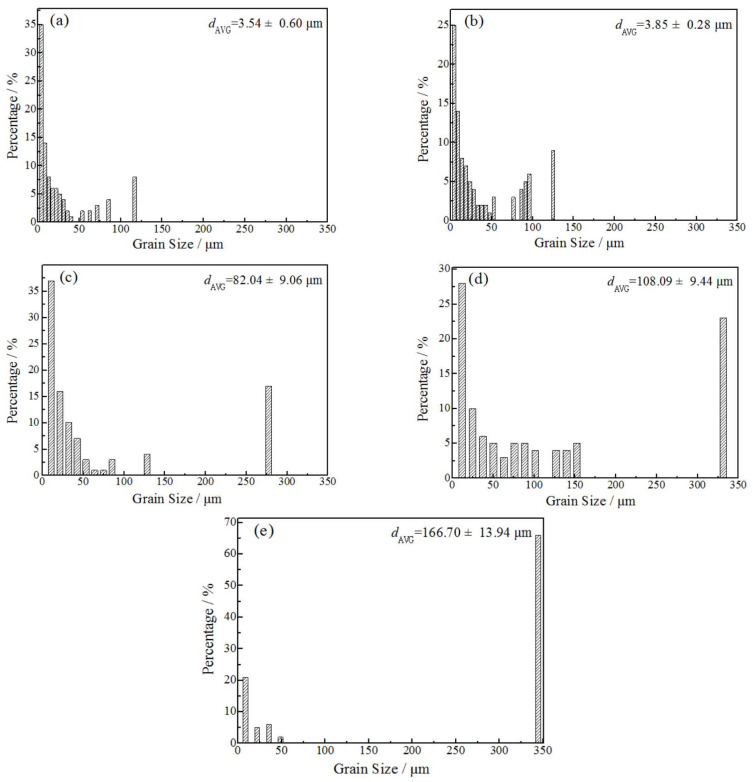
Effect of annealing time on the grain size of rolled and annealed copper foil. (**a**) Rolled sample; (**b**) 1 min; (**c**) 5 min; (**d**) 10 min; (**e**) 60 min.

Figure 6 shows the grain size distribution of the rolled and annealed copper foil. It can be seen that with the increase in annealing time, the average grain size gradually increases, and the grain size distribution range gradually widens. When annealed for more than 10 min, the grain size distribution presents a bimodal structure, which is composed of large recrystallized grains and fine grains. When annealed for 60 min, the percentage of large-size grains is approximately 65%, indicating that the recrystallized grains have coarsened. Compared to the average grain size of samples after annealing from 1 min to 60 min, the coarsening rate of recrystallized grains is more pronounced after annealing from 1 min to 5 min. This difference in the recrystallized grains’ coarsening rate is intimately connected to the HAGBs and LAGBs during the annealing process [18], as shown in Table 1. Meanwhile, this result demonstrates convincingly that the changes in the fraction of HAGBs and LAGBs are closely related to the recrystallization process.

**Figure 7:**
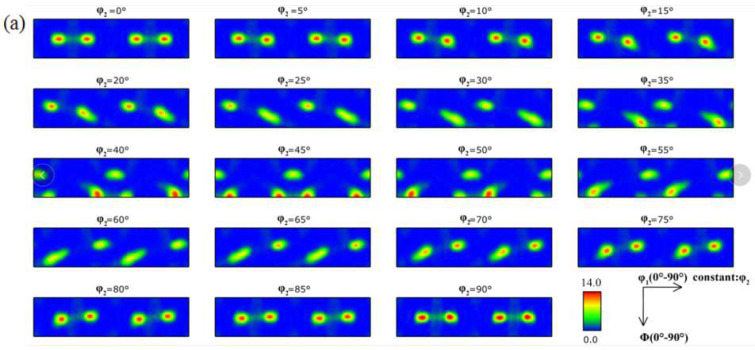

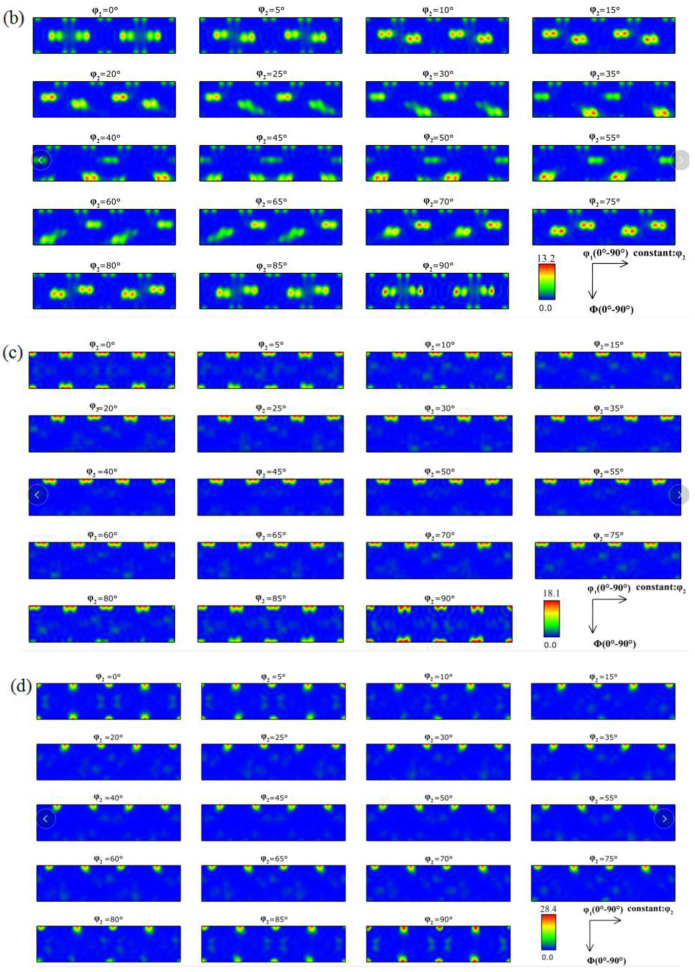

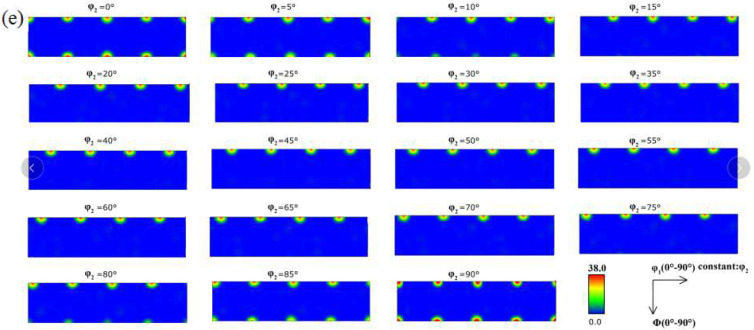
ODF diagram of the rolled and annealed copper foil. (**a**) Rolled sample; (**b**) 1 min; (**c**) 5 min; (**d**) 10 min; (**e**) 60 min.

**Figure 8:**
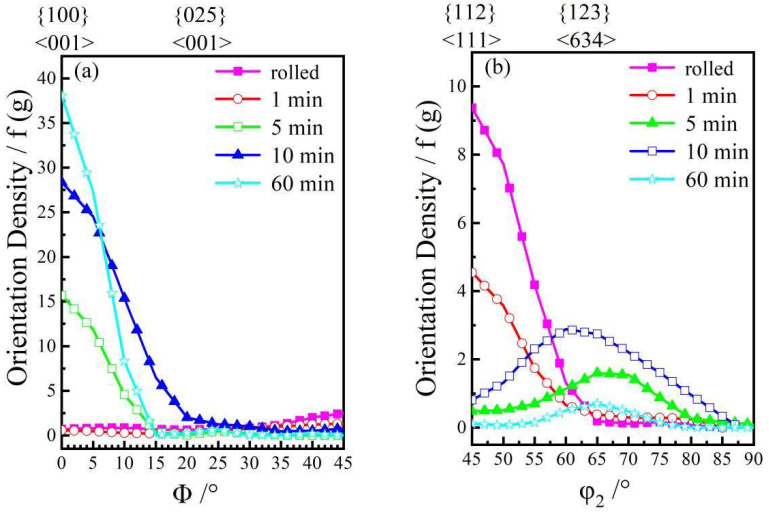

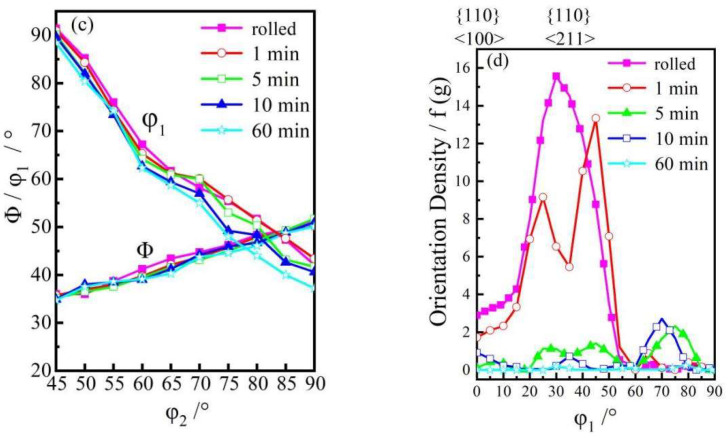
Texture orientation density of the copper foil. (**a**) (0°,0°→45°,0°) fiber; (**b**) β-fiber; (**c**) Position of β-fiber; (**d**) α-fiber.

The texture type and intensity reflect the softening state of copper foil. Figure 7 shows the orientation distribution function (ODF) diagram of the rolled and annealed copper foil. Figure 8 represents texture orientation density along α (0°→90°, 45°, 0°), β (73°, 35°, 45°→90°) and (0°, 0°→45°, 0°) orientation lines, in which, φ_1_ is the angle between the intersection of RD-TD plane and [100]-[010] plane and RD orientation, Φ is the angle between RD-TD plane and [100]-[010] plane, and φ_2_ is the angle between the intersection of RD-TD plane and [100]-[010] plane and [100] orientation. This [100] orientation-[010] orientation-[001] orientation is the crystal coordinate system. From Figure 7a and Figure 8b,d, it can be concluded that the textures of rolled copper foil are copper texture ({112} <111>), brass texture ({110} <211>), S texture ({123} <634>), Gauss texture ({110} <100>), which are typical deformed textures [19], and the maximum texture intensity is 14.0. After annealing for 1 min, the main texture is still deformation texture. Meanwhile, a small amount of cube textures ({100} <001>) and ({025} <001>) begin to appear, which are annealing textures [20]. With the increase in annealing time, the annealing texture intensity increases obviously, and the annealing texture strength reaches 38.0 after annealing for 60 min. Softened annealing will obviously weaken the deformation texture intensity, while significantly enhancing the recrystallization texture intensity.

**Figure 9:**
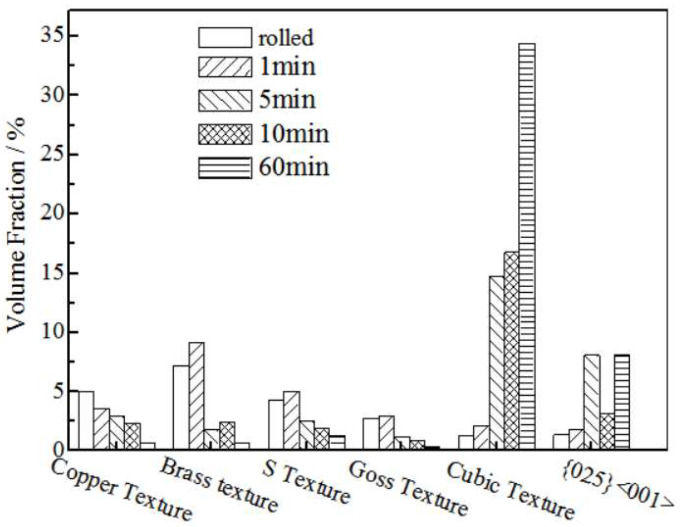
The textures’ fraction of the rolled and annealed copper foil.

Figure 9 shows the textures’ volume fraction of the rolled and annealed copper foil obtained by the orientation density integration method. The rolled copper foil contains 1.0% cubic texture, 1.1% {025} <001> texture, and the brass texture volume fraction is the highest, at 7.2%. After annealing for 1 min, the brass texture and recrystallization texture volume fraction increase. After annealing for 5 min, the deformation texture volume fraction decreases, while the recrystallization texture volume fraction increases significantly. The cubic texture volume fraction is 14.8%, and the {025} <001> texture volume fraction is 8.0%. After annealing for 10 min, the cubic texture volume fraction increases slightly, the {025} <001> texture volume fraction decreases, and the deformation texture volume fraction decreases. After annealing for 60 min, there is almost no deformation texture. Meanwhile, the recrystallization texture volume fraction increases significantly, and the cubic texture volume fraction reaches the highest, at 34.4%. With the increase in annealing time, the copper type texture volume fraction decreases, and the cubic texture volume fraction increases. The main texture of rolled copper foil is the deformation texture, which transfers to the cubic texture and {025} <001> texture in the recrystallization stage and grain growth stage [21].

### 3.2. Mechanical Properties

#### 3.2.1. Tensile Properties

**Figure 10:**
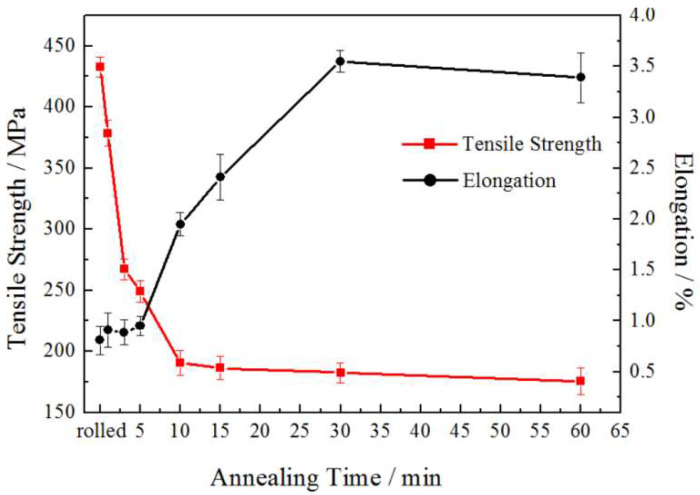
Effect of annealing time on tensile properties of copper foil.

Figure 10 shows the tensile strength and elongation of the rolled and annealed copper foil along the RD orientation. It can be seen that the tensile strength of rolled copper foil is the highest. After annealing for 1–5 min, the tensile strength decreases significantly with the increase in annealing time. After annealing for 10 min, the tensile strength tends to be stable. The reason why the tensile strength decreases rapidly when annealed for 1–5 min is that the LAGBs decrease sharply during the annealing time [22]. The interface energy of the LAGBs is lower than that of the HAGBs, and the mobility of the LAGBs is poor, which weakens the resistance of dislocation movement. Therefore, the tensile strength decreases with the increase in annealing time. After annealing for 5–60 min, the LAGBs fraction changes little, and the tensile strength tends to be stable. When the annealing time increases from 1 min to 30 min, the elongation increases gradually. When the annealing time increases from 30 min to 60 min, the elongation decreases slightly. On the whole, with the increase in annealing time, the elongation of the copper foil increases. This is because with the increase in annealing time, the dislocations obtain enough energy to redistribute. The structural defects, such as the dislocation density, decrease, thus the hindrance to dislocation movement weakens. Therefore, the deformation resistance decreases, the plasticity enhances, and the elongation increases.

#### 3.2.2. Bending Resistance Properties

**Figure 11:**
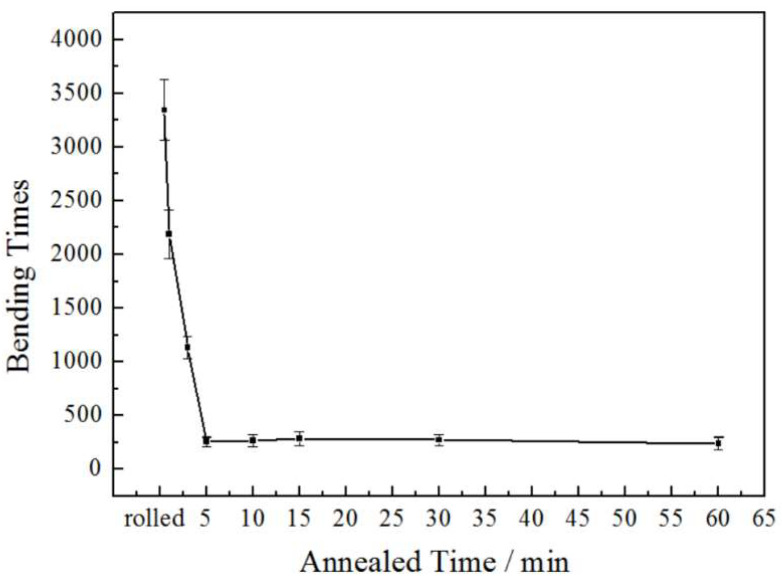
Bending resistance properties of the rolled and annealed copper foil.

Figure 11 shows the effect of annealing time on the bending resistance properties of the rolled and annealed copper foil along the RD orientation. It can be seen that the rolled copper foil has good bending resistance performance, mainly due to the large number of LAGBs, which can effectively inhibit the crack propagation. With the annealing time increasing from 1 min to 5 min, the bending times decreases sharply. The LAGBs fraction decreases sharply with the increase in annealing time. When the annealing time increases from 5 min to 60 min, the bending resistance performance increases slightly. From the analysis of texture evolution, it can be seen that with the increase in annealing time, the deformation texture fraction gradually decreases, while the cubic texture fraction gradually increases. The grain orientation tends to be concentrated, and the crack is not easy to expand. At the same time, the increase in grain size leads to the increase in critical value of crack initiation, which slightly improves the bending resistance properties of the copper foil [23].

**Figure 12:**
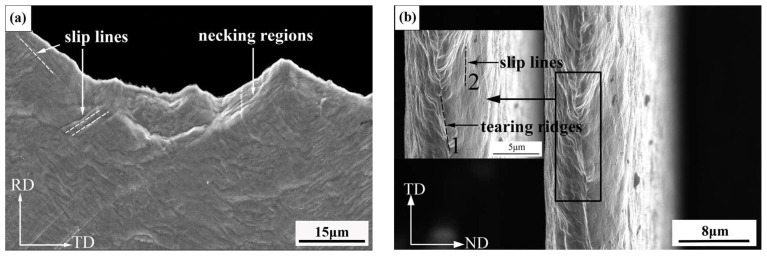
Morphological characteristics of the copper foil annealed for 10 min. (**a**) Surface morphology; (**b**) Fracture morphology.

In order to further analyze the fracture mechanism of the copper foil, the surface morphology and fracture morphology of the longitudinal tensile fracture after annealing for 10 min were studied. As shown in Figure 12, obvious necking phenomenon occurs during the tensile process. When the copper foil is stretched, there will be slip lines, which are 45°to the loading direction, as pointed out with the white dotted line in Figure 12a. The results show that the dislocation movement occurs during the tensile process, and the plastic deformation is mainly carried out by dislocation sliding. From Figure 12b, it can be seen that there are tearing ridges (black dotted line 1) and sliding lines (black dotted line 2) on the tensile fracture surface, showing good plasticity performance.

**Figure 13:**
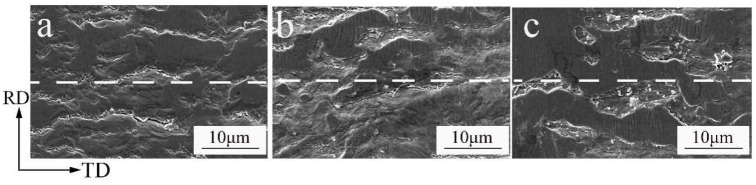
Surface morphology of the copper foil after bending. (**a**) Rolled sample; (**b**) 1 min; (**c**) 3 min.

The fracture surface morphology of the rolled and annealed copper foil after bending test is shown in Figure 13. From Figure 13a, it can be seen that there are different degrees of microcracks on the surface of rolled copper foil, and the direction is parallel to the bending direction. The surface cracks are fine, scattered shallow and flat. After annealing for 1 min, the surface crack depth increases, and some cracks are connected. After annealing for 3 min, the width and depth of the crack increase, and the crack trends to being tortuous. The reason is that there are many LAGBs in the rolled copper foil, and the surface cracks are not easy to concentrate and connect during the bending process, so the bending resistance properties of the rolled copper foil are good [24].

### 3.3. Electrical Performance

**Figure 14:**
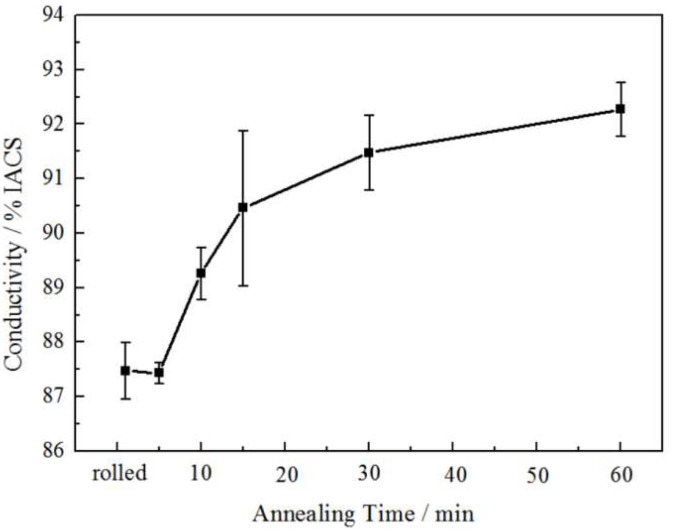
Influence of annealing time on the conductivity of copper foil.

Electrical conductivity is one of the indicators reflecting the change of microstructure. Figure 14 shows the effect of annealing time on the electrical conductivity of the rolled and annealed copper foil. It can be seen that with the increase in annealing time, the electrical conductivity increases from 86% IACS to 92% IACS. During the annealing process, the lattice distortion and crystal defects decrease, the grain size increases, and the grain boundaries decrease. So, the scattering effect of the electrons weakens, the resistivity decreases gradually [25], and electrical conductivity becomes better.

## 4. Discussion

### 4.1. Relationship between the Bending Resistance Properties and Microstructure

Excellent bending resistance performance is one of the most important properties of the rolled copper foil, which is also the premise of wide application [26]. During the deformation process, the grain size, grain orientation, dislocation configuration and dislocation density of the copper foil will change with the deformation rate [27,28]. The bending resistance of the rolled copper foil is obviously better than that of the annealed copper foil, which is due to the fact that the deformation rate of the rolled copper foil is more than 95%. The extra high deformation rate of rolled copper foil will result in smaller grain size and less critical value of bending crack initiation [29]. Meanwhile, the amount of grain boundaries in the same cross-sectional area increases greatly. As an obstacle to the movement of dislocation and other defects, the grain boundaries effectively inhibit the crack propagation and improve the bending resistance performance [30].

It can be seen from Figure 3 that the fraction of <001> oriented grains of the copper foil after annealing for 60 min is 95.2%, showing relatively consistent grain orientation. Grain orientation is also one of the important symbols of bending resistance performance [31]. The more concentrated the preferred orientation of the grains, the smaller the orientation difference between adjacent grains [32,33]. Under certain stress conditions, the difference of Young’s modulus between adjacent grains decreases, and the slip direction tends to be the same, which reduces the difference of plastic deformation of different parts. The stress concentration at the grain boundary will be alleviated, and the initiation and propagation of cracks will also be reduced [34,35]. Therefore, the occurrence of fracture will be delayed, and the bending resistance of copper foil will improve.

### 4.2. Relationship between the Electrical Conductivity and Microstructure

Conductivity, which characterizes the ability of transferring current, is usually related to the temperature, substitution or interstitial atoms, and crystal defects [36]. Because the experiment is carried out at a given temperature, the Ag content and the annealing temperature are also relatively low. The Ag atoms as the replacement atoms would not change during the annealing process, so the electrical conductivity of the copper foil is only related to the defect density [37].

**Figure 15:**
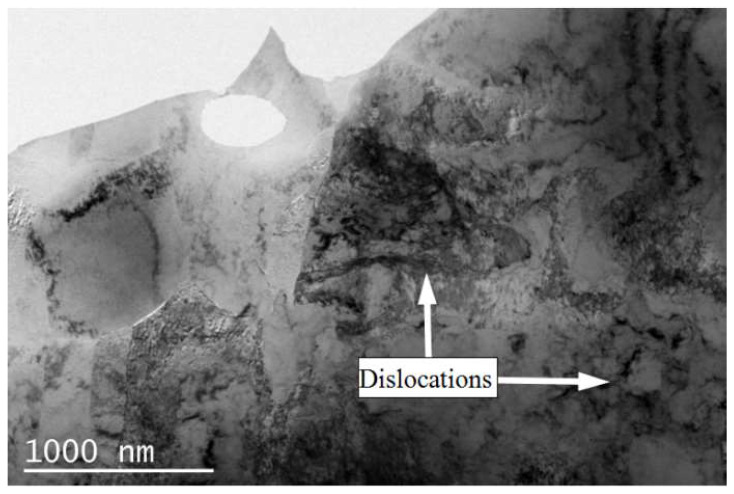
High-density dislocations of the rolled copper foil.

The defect density, especially the dislocation density [38], has an important influence on electrical conductivity. The dislocation density is closely related to the softened annealing time [39]. With the increase in softened annealing time, the dislocation density decreases gradually. According to the two fluid models [40], the moving electrons are scattered and hindered on the dislocations. Meanwhile, the decrease in dislocation density will lead to not only the increase in effective charge density participating in the conduction, but also the enhancement of current transmission ability [41]. Therefore, the electrical conductivity of the copper foil increases with the decrease in dislocation density [42]. The deformation rate of the rolled copper foil is more than 95%, so a large number of dislocations are accumulated in the rolled copper foil (as shown in Figure 15). After the softened annealing, the amount of dislocation defect greatly decreases, and the electrical conductivity increases gradually.

The dislocation density relates to the electrical conductivity and mechanical properties of the copper foil. The mechanical properties reflect the macroscopic properties of materials, while the electrical conductivity mainly explains the change of mechanical properties from the microscopic mechanism [43]. In the past, TEM observation method was often used to analyze the internal structure of metals. This method is not only difficult in terms of preparing the samples and being expensive to detect, but it is also difficult to prepare for quantitative analysis due to the fact that TEM can only observe a small thin area of the sample. Electrical conductivity can be used as a fast and effective method to analyze the internal microstructure of metals.

## 5. Conclusions

Based on the thermal treatment at 180 °C for different times, the softened microstructure and properties of 12 μm thick rolled copper foil were systematically studied. The research result is of great significance to further improve the etching performance, especially the bending resistance performance of rolled copper foil. The main findings are summarized as follows:

(1) When the rolled copper foil is annealed at 180 °C, the microstructure changes regularly with different annealing times. After annealing for 1–10 min, the recrystallization of copper foil occurs. After annealing for 10–60 min, the grains grow, and the annealing texture is mainly cubic texture. With the increase in annealing time, the deformation texture fraction decreases, while the annealing texture fraction increases. When annealed for 10 min, the Σ3 grain boundaries fraction is the highest. The HAGBs fraction increases with the increase in annealing time.
(2) When the rolled copper foil is annealed at 180 °C, the mechanical properties change regularly with different annealing times. After annealing for 1–5 min, the tensile strength decreases sharply because the LAGBs decrease sharply during the annealing time. After annealing for 10–60 min, the tensile strength tends to be stable. The elongation increases with the increase in annealing time. After annealing for 1–5 min, the bending resistance performance decreases significantly. After annealing for 5–60 min, the bending resistance performance improves slightly.
(3) When the rolled copper foil is annealed at 180 °C, the electrical conductivity changes regularly with different annealing times. The electrical conductivity increases gradually with the increase in annealing time. The electrical conductivity of the copper foil reaches 92% IACS after annealing for 60 min. The electrical conductivity can be used as a fast and effective method to analyze the microstructure of metals.

## Figures and Tables

**Table 1:** Effect of annealing time on grain boundary fraction of copper foil.

Annealing Time/min	Rolled	1	5	10	60
LAGBs	68.5%	61.2%	29.1%	24.1%	16.9%
HAGBs	31.5%	38.8%	70.9%	75.9%	83.1%
Σ3 Grain Boundaries	2.7%	4.2%	47.5%	63.3%	57.7%

## Data Availability

The data presented in this study are available on request from the corresponding author.

## References

[1] Xia L., Li Y., Zhao S., Xiong S., Jiang Z. (2020). Corrosion characteristics of rolling oil on the rolled copper foil. Materials.

[2] Wang W., Liu X., Xie J. (2014). Double-coating and porous treatments and evaluation of rolled copper foil surface. Surf. Coat. Technol..

[3] Li J.K., Ren X.P., Zhang Y.L., Hou H.L., Yan Q. (2020). Microstructural response of copper foil to a novel double-cross rolling process. J. Mater. Res. Technol..

[4] Hatano T., Kurosawa Y., Miyake J. (2000). Effect of material processing on fatigue of FPC rolled copper foil. J. Electron. Mater..

[5] Sharma K.P., Mahyavanshi R.D., Kalita G., Tanemura M. (2017). Influence of copper foil polycrystalline structure on graphene anisotropic etching. Appl. Surf. Sci..

[6] Nolden R., Zöll K., Schwarz-Pfeiffer A. (2021). Development of flexible and Functional sequins using subtractive technology and 3D printing for embroidered wearable textile application. Materials.

[7] Yung K.C., Liem H., Choy H.S., Yue T.M. (2010). Impact of plasma etching on fabrication technology of liquid crystal polyer printed circuit board. J. Mater. Sci.-Mater. Electron..

[8] Chiu Y.H., Ou B.L., Lee Y.L. (2017). Effect of thermo-process on anodic capacitor foil manufacturing for AC etching. J. Mater. Sci.-Mater. Electron..

[9] Yoshihara N., Noda M. (2017). Chemical etching of copper foils for single-layer graphene growth by chemical vapor deposition. Chem. Phys. Lett..

[10] Xue S., Wang C., Chen P., Xu Z., Cheng L., Guo B., Shan D. (2019). Investigation of electrically-assisted rolling process of corrugated surface microstructure with T2 copper foil. Materials.

[11] Xiong S., Sun J., Xu Y., Yan X. (2015). Effect of lubricants and annealing treatment on the electrical conductivity and microstructure of rolled copper foil. J. Electron. Mater..

[12] Li J., Ren X., Ling Z., Wang H. (2020). Improving bending property of copper foil by the combination of double-rolling and cross rolling. J. Mater. Res. Technol..

[13] Anand G., Barai K., Madhavan R., Chattopadhyay P.P. (2015). Evolution of annealing texture in cryo-rolled copper. Mater. Sci. Eng. A.

[14] Tseng I., Hsu Y., Leu J., Tu K., Chen C. (2021). Effect of thermal stress on anisotropic grain growth in nano-twinned and un-twinned copper films. Acta Mater..

[15] Chen K.T., Yang Y.C., Yi Y.H., Zheng X.T., Tuan H.Y. (2021). A carbon ink for use in thin, conductive, non peelable, amphiphilic, antioxidant, and large-area current collector coating with enhanced lithium ion battery performance. J. Colloid Interface Sci..

[16] Yadav V., Moelans N., Zhang Y., Jensen D.J. (2021). Influence of geometrical alignment of the deformation microstructure on local migration of grain boundaries during recrystallization: A phase-field study. Scr. Mater..

[17] Zhang H., Zhou H., Qin S., Liu J., Xu X. (2017). Effect of deformation parameters on twinning evolution during hot deformation in a typical nickel-based superalloy. Mater. Sci. Eng. A.

[18] He Q., Jiang X., Cai P., Zhang L., Sun T., Yang X., Zhou K., Zhang L. (2022). Effect of annealing on microstructure and corrosion behavior of interstitial free steel. Materials.

[19] Popova E.N., Deryagina I.L., Valova-Zaharevskaya E.G., Ruello M.L., Popov V.V. (2021). Microstructural Features in Multicore Cu–Nb Composites. Materials.

[20] Ren X., Zhang X., Huang Y., Liu Y., Zhao L., Zhou W. (2020). Evolution of shear texture during the asymmetric rolling and its annealing behavior in a twin-roll casting AA6016 sheet: An ex-situ electron backscatter diffraction study. J. Mater. Res. Technol..

[21] Song M., Liu X., Liu L. (2017). Size effect on mechanical properties and texture of pure copper foil by cold rolling. Materials.

[22] Mishin V.V., Glukhov P.A., Shishov I.A., Stolyarov O.N., Kasatkin I.A. (2019). Structure evolution and mechanical properties of beryllium foils subjected to cold rolling and high-vacuum annealing. Mater. Sci. Eng. A.

[23] Liu X., Li X., Wang X., Xie J. (2014). Relations of rolling reduction and microstructure, texture and bending property of rolled copper foils. Chin. J. Mater. Res..

[24] Chen Q.Z., Jones C.N., Knowles D.M. (2004). The grain boundary microstructures of the base and modified RR 2072 bicrystal superalloys and their effects on the creep properties. Mater. Sci. Eng. A.

[25] Li J., Zhang P., He H., Shi B. (2020). Enhanced the thermal conductivity of flexible copper foil by introducing graphene. Mater. Des..

[26] Girard G., Martiny M., Mercier S. (2020). Experimental characterization of rolled annealed copper film used in flexible printed circuit boards: Identification of the elastic-plastic and low-cycle fatigue behaviors. Microelectron. Reliab..

[27] Chen J.Q., Gao H.T., Hu X.L., Yang L.Q., Ke D.W., Liu X.H., Yan S., Lu R.H., Misra R.D.K. (2020). The significant size effect on nucleation and propagation of crack during tensile deformation of copper foil: Free surface roughening and crystallography study. Mater. Sci. Eng. A.

[28] Zhang J., Chen H., Fan B., Shan H., Chen Q., Jiang C., Hou G., Tang Y. (2021). Study on the relationship between crystal plane orientation and strength of electrolytic copper foil. J. Alloys Compd..

[29] Li X., Li X., Misra R.D.K., Chen Z. (2022). Grain size effect on shearing performance of copper foil: A polycrystal plasticity investigation. Mech. Mater..

[30] Pucillo G.P., Esposito L., Leonetti D. (2019). On the effects of unilateral boundary conditions on the crack growth rate under cycling bending loads. Int. J. Fatigue.

[31] Lezaack M., Hannard F., Simar A. (2022). Understanding the ductility versus toughness and bendability decoupling of large elongated and fine grained Al 7475-T6 alloy. Mater. Sci. Eng. A.

[32] Wang X., Liu X., Xie J. (2014). Mechanism of surface texture evolution in pure copper strips subjected to double rolling. Prog. Nat. Sci.-Mater. Int..

[33] Liao W., Liu X., Yang Y., Wang S., Du M. (2019). Effect of cold rolling reduction rate on mechanical properties and electrical conductivity of Cu-Ni-Si alloy prepared by temperature controlled mold continuous casting. Mater. Sci. Eng. A.

[34] Huang H.L., Ho N.J. (2000). The study of fatigue in polycrystalline copper under various strain amplitude at stage I: Crack initiation and propagation. Mater. Sci. Eng. A.

[35] Paik S., Dutta B.K., Kumar N.N., Tewari R. (2021). Fracture initiation in a single crystal copper edge-crack specimen for various crystallographic orientations. Theor. Appl. Fract. Mec..

[36] Chembarisova R.G. (2020). Influence of grain boundaries on the electrical conductivity of copper alloys. Tech. Phys..

[37] Chen J., Wang X., Gao H., Yan S., Chen S., Liu X., Hu X. (2021). Rolled electrodeposited copper foil with modified surface morphology as anode current collector for high performance lithium-ion batteries. Surf. Coat. Technol..

[38] Dominguez D., Sevostianov I. (2011). Cross-property connection between work-hardening coefficient and electrical resistivity of stainless steel during plastic deformation. Int. J. Fatigue.

[39] Breidi A., Dudarev S.L. (2022). Dislocation dynamics simulation of thermal annealing of a dislocation loop microstructure. J. Nucl. Mater..

[40] Feng R., Li S., Li Z., Tian L. (2012). Variations of microstructure and properties of 690 MPa grade low carbon bainitic steel after tempering. Mater. Sci. Eng. A.

[41] Zhang X., Li H., Zhan M., Zheng Z., Gao J., Shao G. (2020). Electron force-induced dislocations annihilation and regeneration of a superalloy through electrical in-situ transmission electron microscopy observations. J. Mater. Sci. Technol..

[42] Yang F., Dong L., Cai L., Wang L., Xie Z., Fang F. (2021). Effect of cold drawing strain on the microstructure, mechanical properties and electrical conductivity of low-oxygen copper wires. Mater. Sci. Eng. A.

[43] Ablyaz T.R., Shlykov E.S., Muratov K.R., Zhurin A.V. (2022). Study of the EDM process of bimetallic materials using a composite electrode tool. Materials.

